# AMPK promotes lysosomal and mitochondrial biogenesis via folliculin:FNIP1

**DOI:** 10.1093/lifemeta/load027

**Published:** 2023-06-22

**Authors:** Jordana B Freemantle, D Grahame Hardie

**Affiliations:** Division of Cell Signalling & Immunology, School of Life Sciences, University of Dundee, Dundee, DD1 5EH, Scotland, United Kingdom; Division of Cell Signalling & Immunology, School of Life Sciences, University of Dundee, Dundee, DD1 5EH, Scotland, United Kingdom

## Abstract

The AMP-activated protein kinase (AMPK) is known to maintain the integrity of cellular mitochondrial networks by (i) promoting fission, (ii) inhibiting fusion, (iii) promoting recycling of damaged components via mitophagy, (iv) enhancing lysosomal biogenesis to support mitophagy, and (v) promoting biogenesis of new mitochondrial components. While the AMPK targets underlying the first three of these effects are known, a recent paper suggests that direct phosphorylation of the folliculin-interacting protein 1 (FNIP1) by AMPK may be involved in the remaining two.

Cellular networks of mitochondria provide the predominant source of adenosine triphosphate (ATP) in most eukaryotic cells. When and where these networks have become inhibited or damaged, they are fragmented into smaller segments to allow recycling of their components by a type of targeted autophagy termed mitophagy, a process that requires functional lysosomes. Biogenesis of new mitochondrial materials is also required to replace damaged components that have been degraded during mitophagy. Lysosomal biogenesis is primarily induced by the transcription factors transcription factor EB (TFEB) and transcription factor E3 (TFE3) [members of the microphthalmia/transcription factor E (MiT/TFE) family], while the “master regulator” of mitochondrial biogenesis is considered to be the transcriptional coactivator, peroxisome proliferator-activated receptor gamma coactivator 1-alpha (PGC1α). As a signaling pathway switched on by depletion of ATP relative to adenosine diphosphate (ADP) or adenosine phosphate (AMP), the AMP-activated protein kinase (AMPK) system is ideally placed to monitor the output of mitochondria. Moreover, AMPK is already known to trigger fission, and inhibit fusion, of mitochondrial networks via direct phosphorylation of mitochondrial fission factor (MFF) [1] and mitochondrial fission related-1-like (MTFR1L) [2], respectively, as well as promoting mitophagy via phosphorylation of unc-51-like autophagy-activating kinase 1 (ULK1) [3]. However, although connections between AMPK and both lysosomal and mitochondrial biogenesis have previously been proposed, the molecular mechanisms have remained unclear. A recent paper by Malik *et al.* [4] now resolves this by suggesting that AMPK promotes nuclear translocation of TFEB/TFE3, and subsequent expression of a specific splice variant of PGC1α, by phosphorylating multiple sites on folliculin (FLCN)-interacting protein 1 (FNIP1). FNIP1 is a binding partner for FLCN, an activator of the protein kinase mechanistic target-of-rapamycin complex-1 (mTORC1).

The AMPK and mTORC1 signaling pathways, which generally act in direct opposition to each other, both appear to have arisen at a very early stage during eukaryotic evolution [5]. mTORC1, which is activated in cells with abundant supplies of energy and nutrients (especially amino acids), phosphorylates TFEB at three sites, of which two (Ser142 and Ser211) are conserved in TFE3. These phosphorylation events, which can be monitored via a markedly reduced mobility of TFEB or TFE3 during gel electrophoresis, cause their exclusion from the nucleus due to binding of 14-3-3 proteins, which associate with TFEB phosphorylated at Ser211 via a non-canonical binding mode [6]. AMPK is switched on under essentially the opposite circumstances to mTORC1, e.g., during cellular energy stress or starvation for some nutrients. During energy stress, AMPK is activated by the classical or canonical mechanism, in which it senses the ratios of AMP:ATP and/or ADP:ATP via competitive binding of the three adenine nucleotides to a crucial site on its γ subunit [5]. It can also be switched on by starvation for glucose and other carbon sources such as glutamine, via both canonical (AMP-dependent) and non-canonical (AMP-independent) mechanisms [7]. In addition, AMPK is activated by the availability of fatty acids which, following their conversion to Coenzyme A esters, bind to an allosteric site located between the α and β subunits of AMPK, termed the allosteric drug and metabolite (ADaM) site [8]—this site also binds various synthetic activators that have been developed by the pharmaceutical industry, including 991 and MK-8722 (mentioned below). At first sight, activation of AMPK by the presence of nutrients, i.e., fatty acids, might seem counter-intuitive. However, since neither glucose-deprived cells nor cells relying mainly on fatty acids for catabolism can utilize glycolysis as a non-mitochondrial source of ATP, it does make sense that mitochondrial biogenesis should be switched on in the absence of glucose and the presence of fatty acids. These conditions may in fact coincide when fatty acids replace glucose as the principal carbon source for cells, such as in cardiac myocytes during starvation.

FLCN was originally defined in 2002 as the protein encoded by the gene mutated in human Birt-Hogg-Dubé syndrome, which is associated with benign tumors of hair follicles as well as renal malignancies. The paralogs FNIP1 and FNIP2 were identified in 2006 as alternate FNIPs, and FNIP1 was shown to interact with and be phosphorylated by AMPK [9], although the site(s) phosphorylated were not identified at that time. The biochemical functions of the FLCN:FNIP1/2 complexes were unclear for several years, but in 2013, they were reported to carry a GTPase activator protein (GAP) activity toward RagC (or its paralog, RagD), which binds with RagA (or its paralog, RagB) to form a heterodimeric G protein located at the lysosome. This unusual G protein is in the “on” state, which recruits mTORC1 to the lysosome and activates its kinase activity, when the RagA/B partner is in the guanosine triphosphate (GTP)-bound form and the RagC/D partner is in the guanosine diphosphate (GDP)-bound form. The active RagC/D^GDP^ complex is generated by the GAP activity of FLCN:FNIP1, which thus activates mTORC1 [10].

Malik *et al.* [4] began their study by exposing parental and AMPK-α1/-α2 double knockout (DKO) HEK293T cells to various mitochondrial inhibitors, including the Complex I inhibitors rotenone and phenformin, and the uncoupler carbonyl cyanide *m*-chlorophenyl hydrazone (CCCP). Analysis of rapid (2–4 h) changes in gene expression revealed TFEB, or its closely related paralog TFE3, as transcription factors with the most numerous gene targets induced by mitochondrial inhibitors in AMPK wild-type (WT) but not DKO cells. Gene targets for these transcription factors can be recognized by the presence of palindromic sequence motifs, 10 bp in length, in their proximal promoters—these are known as CLEAR (co-ordinated lysosomal expression and regulation) motifs. Mitochondrial genes were also well represented in WT, but not AMPK DKO, cells treated either with mitochondrial inhibitors or the synthetic activator 991.

Based on the original findings that FNIP1 was phosphorylated by AMPK [9] and that the FLCN:FNIP1 complex is an upstream regulator of mTORC1 [10], Malik *et al*. reinvestigated the phosphorylation of FNIP1 and found that AMPK activation in cells caused phosphorylation at five sites, i.e., Ser220, Ser230, Ser232, Ser261, and Ser593, which were all reasonable fits to the AMPK consensus recognition motif. Although the functional effects of phosphorylation of the individual sites were not rigorously examined, mutation of all five of these serine residues to alanine (the SA5 mutant) almost completely eliminated phosphorylation of FLAG-tagged FNIP1 by AMPK in cell-free assays. Therefore, many of their subsequent studies were conducted using FNIP1-depleted HEK293T cells that re-expressed either WT FNIP1 or the SA5 mutant. For example, treatment of WT cells with 991 led to dephosphorylation and nuclear translocation of TFEB (despite the presence of amino acids, which normally activate mTORC1), but this was not seen in SA5-expressing cells. Interestingly, an inhibitory AMPK site on the mTORC1 subunit Raptor (Ser792) was still phosphorylated, and the classical mTORC1 substrates S6K1 and 4EBP1 were still dephosphorylated in SA5-expressing cells treated with 991. This indicates that while phosphorylation of FNIP1 by AMPK affects the regulation of TFEB, it does not affect the regulation of other mTORC1 substrates such as S6K1 and 4EBP. Malik *et al.* [4] also generated a phosphospecific antibody against one of the AMPK sites (Ser220) that was able to detect phosphorylation of endogenous FNIP1 in mouse embryo fibroblasts (MEFs) treated with 991, or in the liver of mice treated *in vivo* with MK-8722, an AMPK activator that was derived from 991 but has better oral availability. In both cases, the Ser220 phosphorylation signal was abolished in MEFs or mouse liver with a double AMPK-α1/-α2 knockout.

Studies with cells expressing GFP-tagged TFEB revealed that treatment with 991 caused dissociation of TFEB from mTOR and Raptor, but increased association with RagC. Purification of lysosomal fractions from cells expressing a tagged lysosomal membrane protein (Tmem192), and fluorescence microscope images probed with labeled antibodies, suggested that RagC dissociated from the lysosome following treatment with 991. In both cases, the effects of 991 were lost in SA5 cells. Additionally, using “GTP-locked” and “GDP-locked” mutants of RagC, they obtained evidence that phosphorylation of FNP1 by AMPK acted by inhibiting its GAP activity (its ability to promote the GTPase activity of RagC), thus converting RagC into its GTP locked, inactive state.

Returning to the effects of AMPK activation on gene expression, treatment of WT and SA5 cells with 991 suggested that around 20% of the AMPK-regulated genes previously observed were dependent on FNIP phosphorylation and that lysosomal genes were highly represented in these. Using a manually curated set of 1500 CLEAR genes, they found that around 75% showed increased transcription after AMPK activation in WT cells, while around 40% did not respond to AMPK activation in SA5 cells. Expression of a few representative CLEAR genes was also monitored by qPCR or western blotting and, in every case, induction by 991 was abolished in the SA5 cells. Many of these gene products were expressed in a biphasic pattern, with a first peak at 1–2 h and a second one at 16–24 h. The lysosomal marker lysosome-associated membrane protein-2 (LAMP2) was used for immunofluorescence imaging, which revealed that both the percentage of lysosomes above a volume threshold (0.1 µm^3^) and the LAMP2 intensity per lysosome increased in response to 991 in WT but not SA5 cells. This supports the idea that AMPK promotes lysosomal biogenesis via the multisite phosphorylation of FNIP1.

Having elucidated how AMPK regulates lysosomal biogenesis, Malik *et al.* [4] next turned their attention to mitochondrial biogenesis. Although there have been various proposals made in recent years as to how AMPK up-regulates PGC1α, most of them have not received independent confirmation. However, Malik *et al*. observed that *PPARGC1A* (encoding PGC1α), an established CLEAR gene, was one of the mRNAs up-regulated in their RNA-seq analysis in WT but not in AMPK knockout or SA5 cells. Intriguingly, however, the *PPARGC1A* gene product appeared to be exclusively a short splice variant of 270 amino acids [n-terminal-PGC1α (NT-PGC1α)], which contains the N-terminal region with its transactivation domain and the two LXXLL-like (L2–L3) motifs that interact with members of the nuclear receptor family such as estrogen-related receptor-α (ERRα) and ERRγ, but lacks binding sites for some other transcription factors and also C-terminal phosphorylation sites that act as triggers for protein degradation. NT-PGC1α is therefore a more stable protein although cooperating with a more limited set of transcription factors. Using western blotting, the group was able to detect the NT-PGC1α protein, but not the full-length variant, after treatment with 991 in WT but not in AMPK knockout or SA5 cells. Studies with cells depleted of PGC1α supported the idea that expression of NT-PGC1α during the early response to AMPK activation (the first wave of gene expression) was ultimately responsible for the increased expression of mitochondrial genes, with the latter tending to occur in the second wave. Analysis of several specific mitochondrial genes by qPCR confirmed that they were induced by AMPK activation in WT but not SA5 cells, mainly in the second wave. As final confirmation that AMPK increased mitochondrial biogenesis via phosphorylation of FNIP1, they showed that 991 treatment of cells for 24 h caused a 30% increase in the abundance of mitochondrial DNA and increased staining using antibodies against the mitochondrial marker isocitrate dehydrogenase-2 (IDH2), neither of which were observed in SA5 cells.

One of the non-mitochondrial genes induced after AMPK activation was *ESRRA* (encoding the nuclear receptor ERRα) and this effect was repressed in cells depleted of PGC1α. Since NT-PGC1α has LXXLL-like binding sites for ERRα, this suggested that cooperation between PGC1α and ERRα was responsible for inducing many mitochondrial genes. This was supported by studies of ERRα knockout cells, in which some representative mitochondrial genes, including *CPT1A*, *COX6A1*, *IDH2*, and *PDHA1*, were not induced by 991.

Overall, this study represents an experimental tour-de-force that clarifies key mechanisms for the regulation of both lysosomal and mitochondrial biogenesis by AMPK (Fig. 1), where the overall effects have been known for several years but the molecular mechanisms have remained obscure. One area where further studies might be useful is in delineating the exact roles of the five individual phosphorylation sites for AMPK on FNIP1. Whilst the mutation of all the five sites was necessary to largely eliminate phosphorylation by AMPK in cell-free assays, this does not prove that all the five are required to regulate the GAP activity of the FLCN:FNIP1 complex. With the five sites, we calculate that there are at least 28 possible combinations of single, double, triple, quadruple, and quintuple mutants and, especially in the absence of a simple cell-free assay for the GAP activity of the FLCN:FNIP1 complex, testing all of them would be a tall order. However, other approaches might be possible. For example, the authors did not address the role of FNIP2, the alternate binding partner for FLCN. While FNIP1 and FNIP2 are closely related paralogs, only three of the AMPK phosphorylation sites in FNIP1 (Ser230, Ser232, and Ser261) are conserved in FNIP2 (as Thr214, Ser216, and Ser244), and Thr214 and Ser216 do not appear to lie within conventional AMPK recognition motifs. It would therefore be interesting to know whether expression of FNIP2 would rescue the effects of 991 on TFEB/TFE3 phosphorylation in the FNIP1 knockout HEK-293T cells that they generated. If it did, this might make identification of the critical sites much easier; if it did not, that would be an interesting result that would imply that FNIP1 and FNIP2 have distinct physiological roles.

**Figure 1:**
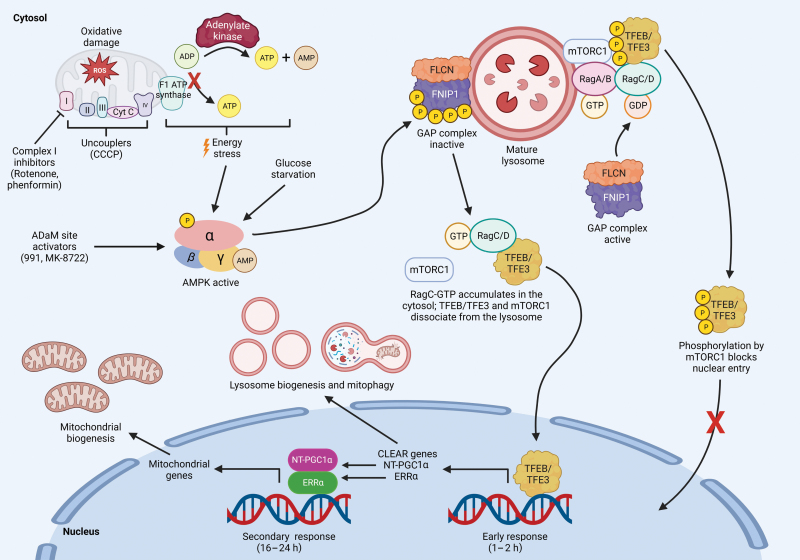
Inhibition of the GAP activity of FLCN:FNIP1 GAP by AMPK induces lysosomal and mitochondrial biogenesis. Activation of AMPK by mitochondrial inhibition or damage, energy stress, allosteric activators that bind the ADaM site, or glucose starvation promotes phosphorylation of FNIP1 at five sites, rendering the GAP activity of the FLCN:FNIP1 complex for RagC/D inactive. RagC/D:GTP then accumulates in the cytosol, reversing the inhibitory phosphorylation of TFEB/TFE3 by mTORC1 and causing dissociation of the latter from the lysosome. Active TFEB/TFE3 translocates to the nucleus to induce the transcription of CLEAR genes promoting lysosome biogenesis and mitophagy (early response) and of NT-PGC1α promoting mitochondria biogenesis (secondary response). P, phosphorylation. (Created with BioRender.com.).

The AMPK system was originally identified by its ability to acutely regulate metabolic enzymes such as acetyl-CoA carboxylase and 3-hydroxy-3-methylglutaryl-CoA reductase, which catalyze key regulatory steps in fatty acid and sterol synthesis, respectively. The study by Malik *et al.* [4] emphasizes that there is much more to AMPK than just acute metabolic regulation, with the biogenesis of key cellular organelles such as lysosomes and mitochondria also being an important part of its remit. Nevertheless, it can be argued that its functions in the maintenance of the cellular mitochondrial networks still fit with the idea that the key role of AMPK lies in cellular energy homeostasis.
